# Supplementary material for: The Systems Biology Research Tool: evolvable open-source software
Source: BMC Syst Biol. 2008 Jun 29;2:55. doi: 10.1186/1752-0509-2-55 (PMC2446383; doi:10.1186/1752-0509-2-55)
Supplement: Additional file 1 — SBRT Archive. An archive of the current version of the Systems Biology Research Tool. [file 1752-0509-2-55-S1.zip › sbrt-1.4.0/doc/developers_guide/api/sbrt/shell/io/class-use/MapOutputFile.html]

Uses of Interface sbrt.shell.io.MapOutputFile


|  |  |  |
| --- | --- | --- |
| **Overview** \| **Package** \| **Class** \| **Use** \| **Tree** \| **Deprecated** \| **Index** \| **Help** | |  |
| PREV   NEXT | **FRAMES**    **NO FRAMES**     **All Classes** |  |


---


## **Uses of Interface sbrt.shell.io.MapOutputFile**

| Packages that use MapOutputFile | |
| --- | --- |
| **sbrt.shell.io** | Provides classes and interfaces for reading and writing files. |
| **sbrt.shell.mng.fba** | Provides classes and interfaces for creating Flux Balance Analysis process managers. |

Uses of MapOutputFile in sbrt.shell.io

| Subinterfaces of MapOutputFile in sbrt.shell.io | |
| --- | --- |
| `interface` | `MapTextOutputFile<K,V>`             This interface is used to represent output files that maps can be written to as text. |

| Classes in sbrt.shell.io that implement MapOutputFile | |
| --- | --- |
| `class` | `AbstractMapOutputFile<K,V>`             This abstract class is a skeleton implementation of `MapTextOutputFile`. |
| `class` | `ComoFile<K,V>`             This class is used to write maps to text files in a column-oriented way. |
| `class` | `RomoFile<K,V>`             This class is used to write maps to text files in a row-oriented way. |
| `class` | `SmoFile<K,V>`             This class is used to write serialized maps to output files. |

Uses of MapOutputFile in sbrt.shell.mng.fba

| Methods in sbrt.shell.mng.fba that return MapOutputFile | |
| --- | --- |
| `static MapOutputFile<java.lang.String,java.lang.Double>` | `InitialPointManager.getCVectorFile(ManagerInput<java.lang.String,java.lang.String> input, java.io.PrintWriter out, Fluxome fluxome)`             Returns an empty output file that is suitable for storing a single C vector with the name denoted by `InitialPointManager.P_VECTOR_FILE_NAME` in the provided input. |
| `static MapOutputFile<java.lang.String,java.lang.Double>` | `SvInitialPointManager.getFluxVectorFile(ManagerInput<java.lang.String,java.lang.String> input, java.io.PrintWriter out, Fluxome fluxome)`             Returns an empty output file that is suitable for storing multiple flux vectors. |
| `static MapOutputFile<java.lang.String,java.lang.Double>` | `InitialPointManager.getFluxVectorFile(ManagerInput<java.lang.String,java.lang.String> input, java.io.PrintWriter out, Fluxome fluxome)`             Returns an empty output file that is suitable for storing a single flux vector. |
| `static MapOutputFile<java.lang.String,java.lang.Double>` | `CdHarFluxVectorManager.getMultiFluxVectorOutputFile(ManagerInput<java.lang.String,java.lang.String> input, java.io.PrintWriter out, Fluxome fluxome)`             Returns an empty output file that is suitable for storing multiple flux vectors. |
| `static MapOutputFile<java.lang.String,java.lang.Double>` | `FbaManager.getMultiFluxVectorOutputFile(ManagerInput<java.lang.String,java.lang.String> input, java.io.PrintWriter out, Fluxome fluxome)`             Returns an empty output file that is suitable for storing multiple flux vectors. |
| `static MapOutputFile<java.lang.String,java.lang.Double>` | `FbaManager.getMultiFluxVectorOutputFile(java.lang.String fileName, FileFormat fileFormat, java.util.Set<java.lang.String> rxnNameHeaders, Fluxome fluxome)`             Returns an empty output file that is suitable for storing multiple flux vectors. |
| `static MapOutputFile<java.lang.String,java.lang.Double>` | `FbaManager.getSingleFluxVectorOutputFile(ManagerInput<java.lang.String,java.lang.String> input, java.io.PrintWriter out, Fluxome fluxome)`             Returns an empty output file that is suitable for storing a single flux vector. |
| `static MapOutputFile<java.lang.String,java.lang.Double>` | `FbaManager.getSingleFluxVectorOutputFile(java.lang.String fileName, FileFormat fileFormat, java.util.Set<java.lang.String> rxnNameHeaders, Fluxome fluxome)`             Returns an empty output file that is suitable for storing a single flux vector. |

---


|  |  |  |
| --- | --- | --- |
| **Overview** \| **Package** \| **Class** \| **Use** \| **Tree** \| **Deprecated** \| **Index** \| **Help** | |  |
| PREV   NEXT | **FRAMES**    **NO FRAMES**     **All Classes** |  |


---
